# Through-Focus Vision Performance and Light Disturbances of 3 New Intraocular Lenses for Presbyopia Correction

**DOI:** 10.1155/2018/6165493

**Published:** 2018-01-31

**Authors:** Santiago Escandón-García, Filomena J. Ribeiro, Colm McAlinden, António Queirós, José M. González-Méijome

**Affiliations:** ^1^Clinical & Experimental Optometry Research Lab (CEORLab), Center of Physics, University of Minho, Braga, Portugal; ^2^Hospital da Luz, Lisboa, Portugal; ^3^Department of Ophthalmology, Glangwili Hospital, Hywel Dda University Hospital Board, Carmarthen, UK; ^4^School of Ophthalmology and Optometry, Wenzhou Medical University, Wenzhou, Zhejiang, China

## Abstract

**Purpose:**

To compare the through-focus visual performance in a clinical population of pseudophakic patients implanted with two new trifocal intraocular lenses (IOLs) and one extended depth of focus IOL.

**Methods:**

Prospective, nonrandomized, examiner-masked case series. Twenty-three patients received the FineVision® and seven patients received the PanOptix™ trifocal IOLs. Fifteen patients received the Symfony extended depth of focus IOL. Mean age of patients was 63 ± 8 years. Through-focus visual acuity was measured from –3.00 to +1.00 D vergences. Contrast sensitivity was measured with and without a source of glare. Light disturbances were evaluated with the Light Distortion Analyzer.

**Results:**

Though-focus evaluation showed that trifocal IOLs performed significantly better at near distance (33 and 40 cm), and extended depth of focus performed significantly better at intermediate distance (1.0 m). Contrast sensitivity function with glare and dysphotopsia was similar between the three IOLs and subjective response to questionnaire showed a significantly higher score (worse performance) for the extended depth of focus IOL compared to both trifocal IOLs in the bothersome subscale (*p* < 0.05).

**Conclusions:**

Trifocal IOLs grant better performance at near distance while extended depth of focus IOL performs better at intermediate distance. Objective dysphotopsia measured with the Light Distortion Analyzer is not reduced in extended depth of focus IOL compared to trifocal IOLs.

## 1. Introduction

Multifocal intraocular lenses (IOLs) are increasingly implanted in pseudophakic patients to increase spectacle independence. In the recent years, the number of new optical designs available has increased. Bifocal and trifocal diffractive IOLs are the most commonly implanted and provide a range of vision from distance to intermediate and near distance. These IOLs allow independence of spectacle correction for intermediate and near vision [1]. However, the simultaneous focusing of objects from different distances across a distance of approximately 1 mm in front of the retina along the optical axis induces formation of haloes and other visual phenomena around the best focused image [2]. Complaints of night vision disturbances or photic phenomena are common with these devices causing some degree of dissatisfaction with the outcomes, which accounts for more than a third of the main reasons for IOL explantation [3, 4].

Considering the potential medical impact of photic phenomena, there have been efforts to investigate and quantify these complaints. Several instruments have been used including halometers [5] and subjective questionnaires [6, 7]. Mastropasqua et al. [8] used some subcategories of the National Eye Institute Refractive Error Quality of Life Instrument-42 (NEI-RQL42) questionnaire comparing three groups of 20 patients each implanted bilaterally with bifocal 2.5 D, bifocal 3.0 D, and mixed contralateral implantation of each lens. Gundersen and Potvin [9] used the National Eye Institute Visual Function Questionnaire (NEI-VFQ) in two cohorts of 11 patients each implanted with diffractive bifocal toric and trifocal toric IOLs. The recently developed Quality of Vision (QoV) questionnaire by McAlinden et al. [10, 11] includes specific categories for dysphotopic phenomena with simulation images and might be a more adequate instrument to subjectively assess the performance of multifocal IOLs.

In a further attempt to get objective metrics of the photic phenomena, different instruments and devices have been engineered. Systems aiming to measure the size of the distortion by analyzing a certain area of the visual field only and expanding the results to 360° of the field usually assume that the distortion might be circular and symmetric in shape [2, 12], which is not actually the case for most people describing such phenomena. More recently, Puell et al. [13] investigated the size of the halo in the general population using the commercial Vision Monitor device. This device measures the ability to recognize letters in three semimeridians around a source of glare at 2.5 m. Indeed, the systems described provide a single value of size around the central source of light. Other experimental devices have been described for use in clinical practice that present peripheral detection stimuli in the form of fluorescent paint which might limit their use as uniform methods for visual assessment [14].

The Light Distortion Analyzer (LDA) is a device consisting of 240 individual light-emitting diodes (LEDs) surrounding a central larger LED acting as source of glare [15]. The exam is performed in a darkened room at 2.0 ms, and it provides different morphological metrics of light disturbances in 30 to 90 seconds per exam [16, 17] which are sensitive to small changes in optical higher-order aberrations [18].

With the aim to reduce the complaints of photic phenomena, the concept of extended depth of focus IOLs has been developed to provide more consistent distance and intermediate vision with less photic phenomena, at the expense of some loss of near vision [19]. Trifocal IOLs have also showed better performance in terms of halo formation compared to bifocal IOLs [20].

Considering the existing concern of photic phenomena with modern IOLs, the potential benefit of new devices in terms of reduction of visual complaints, and the existence of newly developed systems to capture quantitative metrics of such phenomenon, the main goal of the present study is to compare the visual performance of three multifocal IOLs with particular attention to the subjective complaints and the quantitative measurement of the photic phenomena in pseudophakic patients after cataract extraction.

## 2. Material and Methods

This was a prospective study involving patients bilaterally implanted with one brand of multifocal IOL (the same IOL implanted in both eyes). These IOLS were implanted following cataract extraction with phacoemulsification and targeted for emmetropia. Inclusion criteria for enrollment in the present study included no active ocular disease except cataract, nonsevere dry eye, uneventful cataract surgery and postoperative healing process, clear posterior capsule and lens implant, no pupillary abnormality, postop refractive error within ±1.00 diopters (D), and an unaided postoperative visual acuity of 0.10 logMAR or better. Exclusion criteria were IOL dislocation, posterior capsule opacification, or any vitreous or retinal disease. In agreement with the Declaration of Helsinki, the protocol of the study was reviewed and approved by the Ethics Committee of Hospital da Luz (Lisbon, Portugal). Before data collection, patients were instructed on the purpose of the study and procedures used and signed a consent form before formal enrollment.

Surgical procedures were conducted by the same experienced surgeon (FR) under local anesthesia through a microincision of 2.2 mm. Prior to surgery, patients underwent a comprehensive ophthalmological examination including optical biometry and anterior surface optical tomography for the calculation of the power of the IOL using a semicustomized ray-tracing method [21]. Surgical procedures with IOL implantation were conducted with a difference of 7 days between eyes. A summary of the technical details of the IOLs implanted is presented in Table 1.

The AcrySof® IQ PanOptix (TFNT00) is a single-piece copolymer acrylate-methacrylate trifocal IOL (Alcon Laboratories, Texas, USA). The posterior lens surface is spherical, and the anterior surface is aspheric with a diffractive/refractive surface. The lens incorporates a blue-violet filter with an intermediate addition power of +2.17 D and a maximum addition power of +3.25 D. The lens received a CE mark in June 2015.

The FineVision Pod F (PhysIOL, Liège, BE) is a single-piece 25% hydrophilic acrylic ultraviolet (UV) and blue filter trifocal IOL with an intended intermediate addition power of +1.75 D and a maximum addition power of +3.5 D. The optic zone diameter is 6.15 mm and incorporates a diffractive aspheric front surface and a posterior aspheric surface with a negative spherical aberration of −0.11 microns for a 6.0 mm pupil diameter. The IOL claims reduced halo and glare perception under mesopic conditions due to the maximization of distance vision for larger pupils. The lens received a CE mark in February 2010.

The TECNIS® Symfony model ZXR00 (Abbott Medical Optics, Santa Ana, USA) is a biconvex and pupil-independent diffractive IOL combining an achromatic diffractive surface with an echelette design. The achromatic surface is aimed to correct chromatic aberrations and enhance contrast sensitivity. The echelette design which is a specific type of diffraction grating aims to extend the range of vision. Its overall diameter is 13.0 mm and its optical zone diameter is 6.0 mm. The power spectrum available ranges from +5.0 to +34.0 D and incorporates an UV light-absorbing filter. The lens received a CE mark in June 2014.

The main outcome measures were binocular high-contrast visual acuity for different levels of defocus from +1.00 to −3.00 in 0.50 step. The contrast sensitivity function (CSF) with the Functional Acuity Contrast Test® for 1.5, 3.0, 6.0, 12.0, and 18.0 cycles per degree (cpd) under photopic (85 cd/m^2^) and scotopic (5 cd/m^2^) conditions with glare (28 lux–glare II) was evaluated using the Functional Visual Analyzer (FVA, Stereo Optical Company Inc., USA). Subjective quality of vision was assessed with the Quality of Vision (QoV) questionnaire [6, 10, 22]. The questionnaire consists of 10 items with visual pictures to simulate the visual symptoms for the first 7 items and has three subscales: frequency, severity, and bothersome. The questionnaire has been previously developed and validated with the Rasch analysis. The Rasch-scaled scoring is on a 0–100 scale with higher scores indicating worse quality of vision [23]. There are three scores, one for each of the three subscales. Light distortion analysis for size, shape, and regularity of the halo surrounding a source of glare was assessed with a custom-made device (Light Distortion Analyzer, CEORLab-University of Minho, Portugal). The characteristics of this device, examination routines, and main outcome measures have been previously described and validated in clinical populations [15–17] including pseudophakic patients [20, 24]. The size of the light distortion compared to the total area under evaluation, also known as the light distortion index (LDI%), was calculated. Considering the symmetric bilateral implantation, for monocular analysis, only the right eye was considered. Binocular summation was calculated as the % decrease or increase in light distortion under binocular conditions compared to the average monocular value. Patients were evaluated once between 1 and 3 months after surgery.

Statistical analysis was conducted using SPSS v21.0 (IBM Inc., IL). Normality of data distribution was assessed using the Shapiro-Wilk test. Comparison between monocular and binocular values was evaluated by paired sample *t*-test or nonparametric Wilcoxon signed-rank test, while comparisons between clusters of patients with different IOLs implanted were evaluated with independent sample *t*-test or the nonparametric Mann-Whitney test. Correlations were assessed using Pearson correlation or nonparametric Spearman correlation. ANOVA or Kruskal-Wallis with multiple post-hoc comparisons was used to compare the outcomes among different IOL groups. The level of statistical significance has been set at *p* < 0.050.

## 3. Results

Demographic data of patients enrolled in each group are presented in Table 2 along with pre- and postoperative clinical data. There was a statistically significant difference in J0 between the three groups postoperatively (Kruskal-Wallis test), but the differences were not clinically different. Comparisons for all other parameters indicated no statistically significant differences between groups (*p* > 0.050).

Postoperative uncorrected distance visual acuity was 0.08 ± 0.12 logMAR for the whole sample, and there were no statistically significant differences between the IOL groups (*p* = 0.780). Best-corrected distance visual acuity improved to −0.16 ± 0.27 logMAR for the whole group.

**Figure 1:**
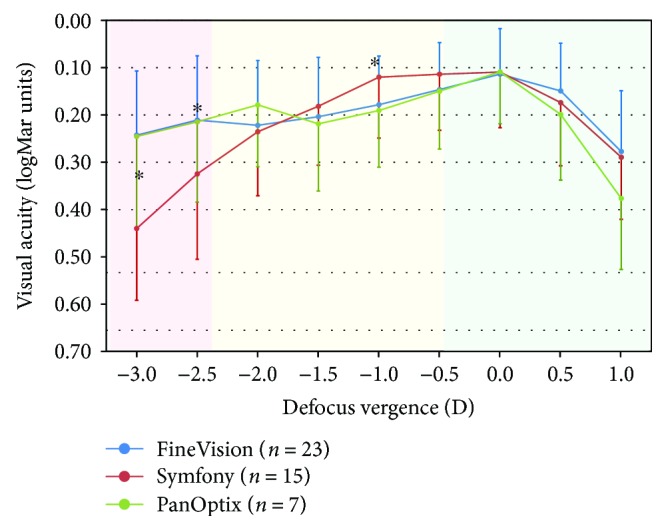
Defocus curves for the three lenses under comparison in this study. Error bars represent 1 × SD. ^∗^Statistically significantly different at 0.05 level (Kruskal-Wallis).

Figure 1 presents the defocus curves for the three lenses under comparison. The three lenses performed similarly for all vergences with the exception of intermediate vision at −1.00 D/1 m (*p* = 0.030) and near vision at −2.5 D/0.4 m (*p* = 0.007) and −3.0 D/0.33 m (*p* = 0.014). The extended depth of focus (EDOF) IOL (Symfony) provided a range of stable maximum visual acuity from infinity to approximately 1 m, dropping almost 1 line in visual acuity (0.18 logMAR) at 67 cm and to 0.44 logMAR at 33 cm. Conversely, the two trifocal IOLs (FineVision and PanOptix) showed a similar behavior, with a worse intermediate vision at 1 m compared to the EDOF IOL being 0.12 logMAR for Symfony and 0.18-0.19 logMAR for FineVision and PanOptix, respectively (*p* < 0.050). The three lenses showed a similar behavior between 67 and 50 cm (*p* > 0.050), and at near vision, both trifocal IOLs showed significantly better performance compared with EDOF IOL (*p* < 0.050).

**Figure 2:**
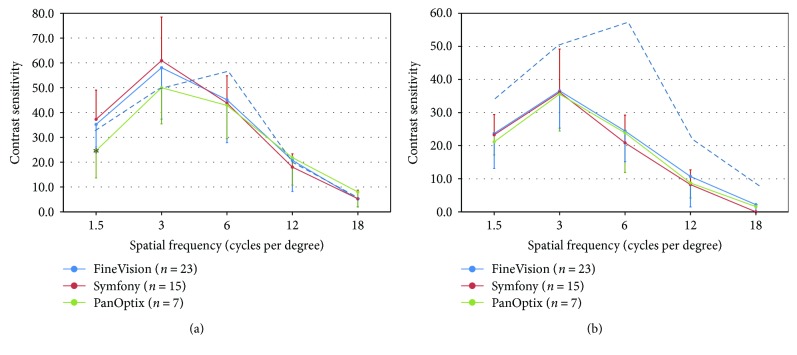
Contrast sensitivity function under photopic (a) and scotopic (b) conditions measured with the Functional Visual Analyzer. Error bars represent 1 × SD. ^∗^Statistically significantly different at 0.05 level (Kruskal-Wallis). To avoid collapsing the lines, only the lower limit of normality is shown (dashed line).

Contrast sensitivity function under photopic and scotopic conditions is presented in Figure 2. Differences between IOLs were not significantly different at any spatial frequency under both conditions (*p* > 0.050). Under photopic conditions (Figure 2(a)), the three IOLs are above the inferior limit of normality for 3, 12, and 18 cpd. Conversely, the three lenses dropped below the inferior limit of normality for 6 cpd, and the PanOptix was below the inferior limit for 1.5 cpd. Only for the lowest frequency, the PanOptix lens performed worse than the other two lenses (*p* = 0.049).

**Figure 3:**
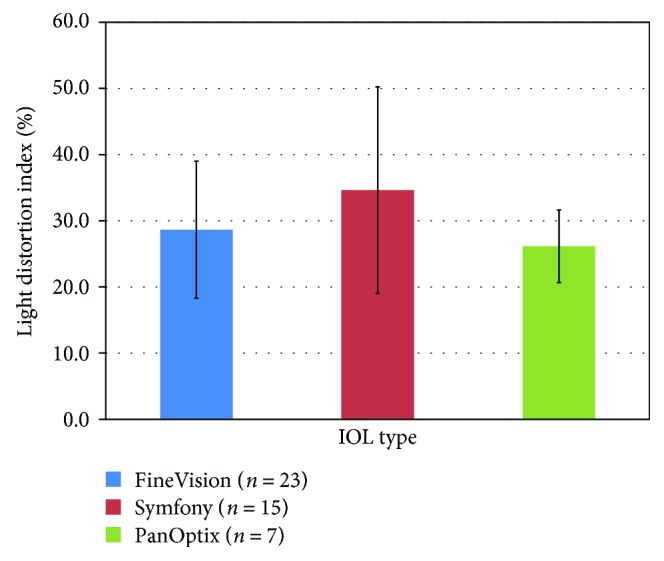
Light distortion index (%) for the three IOLs under evaluation. Error bars represent 1 × SD.

The light distortion analysis (Figure 3) showed that the EDOF IOL had larger values of LDI (34.6 ± 16.0) compared with the two trifocal IOLs but this difference was not statistically significant (*p* = 0.237).

**Figure 4:**
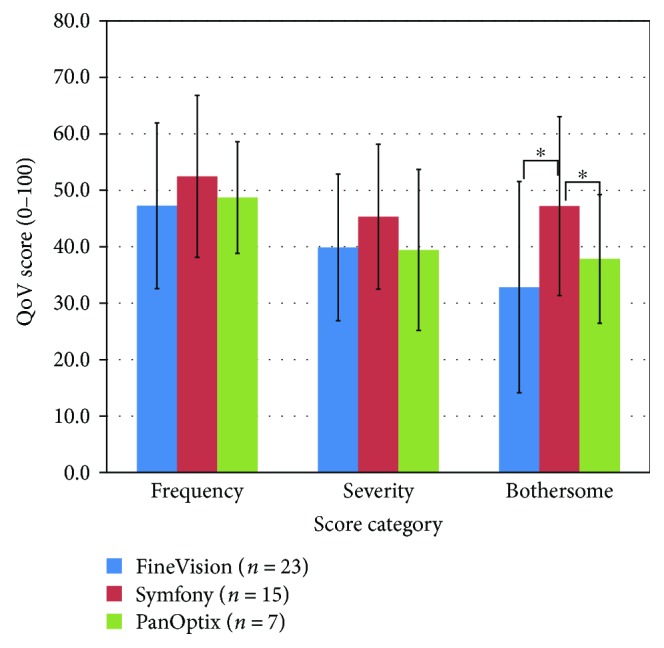
Quality of Vision (QoV) questionnaire scores for the three IOL groups across the three subscales of the questionnaire (frequency, severity, and bothersome). Error bars represent 1 × SD. ^∗^Statistically significantly different at 0.05 level (Kruskal-Wallis).

The results of the subjective questionnaire are presented in Figure 4. While the Symfony IOL presented higher values showing worse performance in all categories, differences were only statistically significant (*p* = 0.011) for the bothersome subscale that reached an average value of 47.2 ± 16.0 compared with that of FineVision (32.8 ± 16.0) or PanOptix (37.9 ± 12.0).

## 4. Discussion

In the present study, we report comparative performance of the three newly designed IOLs for the correction of presbyopia in patients successfully implanted and with optical distance vision correction as shown in the through-focus curves. For distance vision, our uncorrected visual acuity was inferior to the values reported by other authors that might be related to the residual refractive errors reported in Table 2. Discrepancies with the outcomes of other studies [25, 26] might be also related to the methodologies used to record acuity using targets at infinity in devices such as the Functional Visual Analyzer as we used or real targets at shorter distances as the ETDRS charts. However, several other studies do not report the tests used that makes it difficult to compare the results. Visual performance at different vergences showed a similar behavior for both trifocal lenses, as expected. In contrast, the EDOF lens provided a consistent visual performance from distance and intermediate distance, worsening below the performance of the trifocal lenses for 50 cm and closer distances. The uniform range of vision near the 0 vergence might imply that this lens is more robust to errors in the power calculation or in the final position of the lens, without reducing distance vision significantly. This is in disagreement with results presented recently by Gatinel and Loicq [27] reporting optical bench measurements in the Symfony EDOF IOL compared with a bifocal and a trifocal IOL that predict a drop in modulation transfer function (MTF) for the EDOF lens at intermediate vision for pupil sizes larger than 2 mm. Considering that the pupil size of our sample (4.35 mm) is larger than the 3.75 mm of the maximum pupil evaluated by Gatinel and Loicq, a worse performance at intermediate vision would be expected based on optical bench measurements. Our clinical results are much better than those predicted. On the other hand, the results of Domínguez-Vicent predict a better performance for the EDOF lens at 50 cm (vergence +2.00 D) compared with 100 cm (vergence +1.00 D). Our clinical results showing good performance for distance and intermediate vision up to 100 and 70 cm do not agree with previous experimental predictions based on simulation analysis. For example, Gatinel and Loicq [27] obtained a maximum peak of performance based on MTF values at 70 cm accordingly, while Domínguez-Vicent et al. [19] found the best intermediate performance for 50 cm. These differences might be related to the fact that in vitro measurements are obtained with monochromatic light under nonrealistic conditions compared to clinical measurements and the adaptation effects that the patient might undergo over time. Comparing the trifocal IOLs, the PanOptix IOL shows a second peak of improved visual acuity at +2.00 D vergence compared with the FineVision. This might be explained by the intermediate addition provided in the PanOptix (+2.17 D) compared to the FineVision (+1.75 D). This provides a wide range of good vision for the PanOptix from distance to 40 cm as we report, and this is in agreement with preliminary data reported by Kohnen [28] on the first six eyes implanted with this lens. The previous should not be understood as a direct justification of the visual outcomes found in this study as the MTF has shown to be not a good predictor of the clinical visual acuity outcomes [29].

The present study showed also for the first time for the IOLs investigated that the light distortion, as measured with the Light Distortion Analyzer, affects a significant proportion of patients, showing an increase compared to the mean and median values of approximately 15–23% previously reported for monofocal IOLs [20, 24]. These monofocal IOL studies were for older populations (64–70 years of age) compared to the present study (62-63 years). This is in agreement with the common clinical knowledge and also with the preliminary data recently published by Brito et al. [20] with the AT Lisa bifocal and trifocal IOLs. In their investigation of light distortion with the trifocal AT Lisa 839M and bifocal AT Lisa 909MP, the authors reported an average binocular LDI of 29.29% and 40.49%, respectively, compared with 15.28% for the monofocal control group. Our results for the trifocal IOLs (PanOptix and FineVision) agree with those reported by Brito et al. In contrast with the expected, the EDOF IOL showed higher average values compared with the trifocal IOLs.

Despite the fact that average values of light distortion were higher in patients with poorer low-contrast visual acuity and lower contrast sensitivity postoperatively, the correlations between light distortion and the remaining visual functions were generally poor (correlation coefficient < 0.400). This suggests that the CSF and LDA measure different aspects of quality of vision in patients implanted with multifocal IOLs. In IOL patients, contrast sensitivity may be reduced due to decreased visual quality, residual refractive error, split of light into different foci, or intraocular light scatter. In the present study, we found CS values within the normal range expected for the age of the patients under photopic conditions. However, under scotopic conditions with glare, the CS was reduced for all lenses. The three lenses performed similarly in terms of contrast sensitivity. This might be explained by the fact that we measured CSF at distance where the three lenses show accurate refractive outcomes as seen in the defocus curves. The lower values for the PanOptix at lower frequencies compared with the other two lenses might be related to the limited sample in this group compared with the other two.

Mastropasqua et al. evaluated the patient satisfaction after bilateral implantation and combination of two similar multifocal IOLs using the National Eye Institute Refractive Error Quality of Life Instrument-42 questionnaire. Though this questionnaire has not been specifically devised to evaluate dysphotopic phenomena and contains some serious psychometric flaws [30, 31], it includes some questions such as glare. While the 2.5 D addition subgroup showed a higher glare score (better performance) compared with the higher addition, this was not statistically significant [8]. Our results with the QoV questionnaire agree with those with the LDA measurements showing a slightly worse performance with the Symfony compared with the trifocal IOLs, and this difference was statistically significant for the bothersome subscale. As said, this was not expected as this IOL should reduce halo perception. However, in vitro measurements obtained by Gatinel and Loicq show that this lens is expected to show a more intense first halo compared with other trifocal and bifocal lenses. They also report the spherical aberration for this lens and show that for a 4.5 mm pupil (similar to the one shown by our patients), the lens will induce high negative spherical aberration (−0.24 micron). In a recent study, Macedo-de-Araújo et al. [18] showed that inducing positive or negative spherical aberration in a nonaccommodating eye will increase significantly the light distortion size. This negative spherical aberration is necessary to create the EDOF effect, but the consequence might be a larger halo perception under night vision conditions. In contrast, according to Table 1, the remaining two lenses induce a slightly negative spherical aberration that counterbalances the aberration of the cornea in the pseudophakic patient and improves quality of vision. Therefore, we hypothesize that the increase in light distortion in the trifocal lenses is due to the superposition of the near, intermediate, and distance focused and defocused images in the retinal plane, while with the EDOF lens, the increase in negative spherical aberration along with the diffractive optics for the achromatizing purpose that might create some scattering phenomena might add to each other to generate a larger distortion under the conditions of our measurements with this experimental device. This observation is also supported by the QoV questionnaire results, particularly for the bothersome score. In trifocal IOLs, this phenomenon can be explained by the superposition of the near and distance foci [32] but the same mechanism should not explain the findings in the extended depth of focus IOL. The potential involvement of scattered light in the echelletes of the diffractive achromatizing surface is a hypothesis that should be investigated in future studies.

One limitation of the present study is the limited sample. The sample might also be underpowered to detect differences in the light distortion and the subjective QoV questionnaire. This is even more relevant in the PanOptix group with only 7 subjects. Assuming that one of the IOLs under comparison would be hypothesized not to induce haloes, this small sample should warrant 80% statistical power to detect differences in light disturbance of 30 units between lenses on a parallel study design. Instead, we found that all IOLs under comparison induce similar light disturbance as measured with this experimental device with a nonstatistically significant trend for the EDOF IOL to show a worse performance in terms of light distortion and subjective performance (statistically significant in the bothersome domain). Residual refractive errors might also justify differences in performance in terms of light disturbances and visual complaints, but we have not observed significant differences in this domain either in the spherical equivalent or in the astigmatic components (see Table 2). However, even under good refractive outcomes, it is expected that the complaints of dysphotopsia can be present. In a recent large study involving several thousands of patients, nearly 40% of the patients complained of worse night vision after implantation, despite their good uncorrected distance visual acuity [26]. A recent study showed that the first halo estimated in an optical bench for the Symphony lens was more intense compared with that for the trifocal FineVision and a bifocal IOL [23]. This should be further investigated in future studies involving larger sample sizes, including the potential relationship between the size and intensity of the haloes measured in an optical bench and those measured after lens implantation with a psychophysical method such as the LDA used in this work.

## Figures and Tables

**Table 1:** Technical specifications of the IOLs implanted.

	PanOptix	Symfony	FineVision
Technology/design	Trifocal	Extended depth of focus	Trifocal
Diffractive area	4.50 mm	6.00 mm	6.15 mm
Geometry of central zone	Diffractive	Aspheric anterior surface/posterior achromatic diffractive surface	Diffractive
Optic type	Nonapodized	Nonapodized	Apodized
Refractive index	1.55	1.47	1.46
Near add powers in the IOL plane and spectacle plane	3.25 D (2.6 D)	—	3.50 D (2.8 D)
Intermediate add powers in the IOL plane and spectacle plane	2.17 D (1.74 D)	—	1.75 D (1.4 D)
Spherical aberration	−0.10 *μ*m	−0.27 *μ*m	−0.10 *μ*m
Material	Copolymer acrylate-methacrylate	UV-blocking hydrophobic acrylic	26% hydrophilic acrylic
Lens color	Yellow	No pigment	Yellow

**Table 2:** Preoperative demographic data (mean ± SD) of the patients enrolled in this study.

	PanOptix	Symfony	FineVision	Significance (*p*)^†^
Number of patients	7	15	23	
Male : female	1 : 6	2 : 13	7 : 16	
Age ± SD (years)	62.3 ± 9.0	63.5 ± 9.4	62.6 ± 8.0	0.746
Axial length	23.2 ± 0.6	23.2 ± 1.7	24.0 ± 4.4	0.822
IOL power (D)^∗^	21.6 ± 1.4	22.9 ± 4.2	22.0 ± 3.4	0.499
M preoperatively	−0.71 ± 3.21	0.80 ± 3.85	0.96 ± 2.21	0.891
J0 preoperatively	−0.06 ± 0.36	0.20 ± 0.43	−0.19 ± 0.46	0.069
J45 preoperatively	−0.04 ± 0.11	0.01 ± 0.38	−0.01 ± 0.12	0.640
Time ± SD since surgery (days)	42 ± 29	39 ± 13	50 ± 20	0.145
Distance binocular UCVA (postoperatively)	0.07 ± 0.10	0.08 ± 0.10	0.08 ± 0.09	0.780
Distance binocular BSCVA (postoperatively)	−0.07 ± 0.19	−0.10 ± 0.19	−0.24 ± 0.14	0.613
Pupil diameter (mm)	4.6 ± 1.5	4.7 ± 1.3	4.9 ± 1.5	0.406
M postoperatively	0.13 ± 0.24	0.02 ± 0.80	0.27 ± 0.86	0.178
J0 postoperatively	−0.06 ± 0.34	−0.11 ± 0.48	−0.09 ± 1.12	**0.022**
J45 postoperatively	−0.05 ± 0.18	−0.03 ± 0.48	−0.09 ± 1.12	0.891

SD: standard deviation; IOL: intraocular lens; UCVA: uncorrected distance visual acuity; BSCVA: best distance spectacle-corrected visual acuity; M: spherical equivalent; J0 and J45: horizontal and oblique components of the vector decomposition of cylindrical refraction. ^∗^Right eye only (interocular difference ≤ 1.00 D). ^†^Kruskal-Wallis Test.

## References

[1] de Vries N. E., Nuijts R. M. M. A. (2013). Multifocal intraocular lenses in cataract surgery: literature review of benefits and side effects. *Journal of Cataract & Refractive Surgery*.

[2] Pieh S., Lackner B., Hanselmayer G. (2001). Halo size under distance and near conditions in refractive multifocal intraocular lenses. *The British Journal of Ophthalmology*.

[3] Kamiya K., Hayashi K., Shimizu K. (2014). Multifocal intraocular lens explantation: a case series of 50 eyes. *American Journal of Ophthalmology*.

[4] de Vries N. E., Webers C. A. B., Touwslager W. R. H. (2011). Dissatisfaction after implantation of multifocal intraocular lenses. *Journal of Cataract & Refractive Surgery*.

[5] Buckhurst P. J., Naroo S. A., Davies L. N. (2015). Tablet app halometer for the assessment of dysphotopsia. *Journal of Cataract & Refractive Surgery*.

[6] Khadka J., McAlinden C., Pesudovs K. (2013). Quality assessment of ophthalmic questionnaires: review and recommendations. *Optometry and Vision Science*.

[7] Cillino G., Casuccio A., Pasti M., Bono V., Mencucci R., Cillino S. (2014). Working-age cataract patients: visual results, reading performance, and quality of life with three diffractive multifocal intraocular lenses. *Ophthalmology*.

[8] Mastropasqua R., Pedrotti E., Passilongo M., Parisi G., Marchesoni I., Marchini G. (2015). Long-term visual function and patient satisfaction after bilateral implantation and combination of two similar multifocal IOLs. *Journal of Refractive Surgery*.

[9] Gundersen K. G., Potvin R. (2016). Comparison of visual outcomes after implantation of diffractive trifocal toric intraocular lens and a diffractive apodized bifocal toric intraocular lens. *Clinical Ophthalmology*.

[10] McAlinden C., Pesudovs K., Moore J. E. (2010). The development of an instrument to measure quality of vision: the Quality of Vision (QoV) questionnaire. *Investigative Ophthalmology & Visual Science*.

[11] McAlinden C., Skiadaresi E., Pesudovs K., Moore J. E. (2011). Quality of vision after myopic and hyperopic laser-assisted subepithelial keratectomy. *Journal of Cataract & Refractive Surgery*.

[12] Lackner B., Pieh S., Schmidinger G. (2003). Glare and halo phenomena after laser in situ keratomileusis. *Journal of Cataract & Refractive Surgery*.

[13] Puell M. C., Perez-Carrasco M. J., Barrio A., Antona B., Palomo-Alvarez C. (2013). Normal values for the size of a halo produced by a glare source. *Journal of Refractive Surgery*.

[14] Kojima T., Hasegawa A., Hara S. (2011). Quantitative evaluation of night vision and correlation of refractive and topographical parameters with glare after orthokeratology. *Graefe's Archive for Clinical and Experimental Ophthalmology*.

[15] Linhares J. M. M., Neves H., Lopes-Ferreira D., Faria-Ribeiro M., Peixoto-de-Matos S. C., González-Méijome J. M. (2013). Radiometric characterization of a novel led array system for visual assessment. *Journal of Modern Optics*.

[16] Ferreira-Neves H., Macedo-de-Araújo R., Rico-del-Viejo L., da-Silva A. C., Queirós A., González-Méijome J. M. (2015). Validation of a method to measure light distortion surrounding a source of glare. *Journal of Biomedical Optics*.

[17] Santolaria E. S., Cerviño A., Queiros A., Villa-Collar C., Lopes-Ferreira D., González-Méijome J. M. (2015). Short-term changes in light distortion in orthokeratology subjects. *BioMed Research International*.

[18] Macedo-de-Araújo R., Ferreira-Neves H., Rico-del-Viejo L., Peixoto-de-Matos S. C., González-Méijome J. M. (2016). Light distortion and spherical aberration for the accommodating and nonaccommodating eye. *Journal of Biomedical Optics*.

[19] Domínguez-Vicent A., Esteve-Taboada J. J., Del Águila-Carrasco A. J., Ferrer-Blasco T., Montés-Micó R. (2016). In vitro optical quality comparison between the Mini WELL Ready progressive multifocal and the TECNIS Symfony. *Graefe's Archive for Clinical and Experimental Ophthalmology*.

[20] Brito P., Salgado-Borges J., Neves H., Gonzalez-Meijome J., Monteiro M. (2015). Light-distortion analysis as a possible indicator of visual quality after refractive lens exchange with diffractive multifocal intraocular lenses. *Journal of Cataract & Refractive Surgery*.

[21] Ribeiro F. J., Castanheira-Dinis A., Dias J. M. (2012). Personalized pseudophakic model for refractive assessment. *PLoS One*.

[22] McAlinden C., Skiadaresi E., Gatinel D., Cabot F., Huang J., Pesudovs K. (2013). The Quality of Vision questionnaire: subscale interchangeability. *Optometry and Vision Science*.

[23] Skiadaresi E., McAlinden C., Pesudovs K., Polizzi S., Khadka J., Ravalico G. (2012). Subjective quality of vision before and after cataract surgery. *Archives of Ophthalmology*.

[24] Salgado-Borges J., Dias L., Costa J., Ferreira-Neves H., Peixoto-de-Matos S. C., González-Méijome J. M. (2015). Light distortion and ocular scattering with glistening and aberration-free pseudophakic IOL: a pilot study. *Journal of Emmetropia*.

[25] Attia M. S. A., Auffarth G. U., Kretz F. T. A. (2017). Clinical evaluation of an extended depth of focus intraocular lens with the Salzburg reading desk. *Journal of Refractive Surgery*.

[26] Bilbao-Calabuig R., Llovet-Rausell A., Ortega-Usobiaga J. (2017). Visual outcomes following bilateral lmplantation of two diffractive trifocal intraocular lenses in 10 084 eyes. *American Journal of Ophthalmology*.

[27] Gatinel D., Loicq J. (2016). Clinically relevant optical properties of bifocal, trifocal, and extended depth of focus intraocular lenses. *Journal of Refractive Surgery*.

[28] Kohnen T. (2015). First implantation of a diffractive quadrafocal (trifocal) intraocular lens. *Journal of Cataract & Refractive Surgery*.

[29] Alarcon A., Canovas C., Rosen R. (2016). Preclinical metrics to predict through-focus visual acuity for pseudophakic patients. *Biomedical Optics Express*.

[30] McAlinden C., Skiadaresi E., Moore J., Pesudovs K. (2011). Subscale assessment of the NEI-RQL-42 questionnaire with Rasch analysis. *Investigative Ophthalmology & Visual Science*.

[31] McAlinden C., Khadka J., de Freitas Santos Paranhos J., Schor P., Pesudovs K. (2012). Psychometric properties of the NEI-RQL-42 questionnaire in keratoconus. *Investigative Ophthalmology & Visual Science*.

[32] Alba-Bueno F., Garzón N., Vega F., Poyales F., Millán M. S. (2017). Patient-perceived and laboratory-measured halos associated with diffractive bifocal and trifocal intraocular lenses. *Current Eye Research*.

